# Tunable reflecting terahertz filter based on chirped metamaterial structure

**DOI:** 10.1038/srep38732

**Published:** 2016-12-12

**Authors:** Jing Yang, Cheng Gong, Lu Sun, Ping Chen, Lie Lin, Weiwei Liu

**Affiliations:** 1 Institute of Modern Optics, Nankai University, Key Laboratory of Optical Information Science and Technology, Ministry of Education, Tianjin 300350, China; 2 Cooperative Innovation Centre of Terahertz Science, No.4, section 2, North Jianshe Road, Chengdu 610054, China

## Abstract

Tunable reflecting terahertz bandstop filter based on chirped metamaterial structure is demonstrated by numerical simulation. In the metamaterial, the metal bars are concatenated to silicon bars with different lengths. By varying the conductivity of the silicon bars, the reflectivity, central frequency and bandwidth of the metamaterial could be tuned. Light illumination could be introduced to change the conductivity of the silicon bars. Numerical simulations also show that the chirped metamaterial structure is insensitive to the incident angle and polarization-dependent. The proposed chirped metamaterial structure can be operated as a tunable bandstop filter whose modulation depth, bandwidth, shape factor and center frequency can be controlled by light pumping.

Metamaterials provide new opportunities to realize the tailored interactions with terahertz (THz) waves, which are often unavailable or very difficult to obtain from natural materials^1^,^2^,^3^. A few interesting and useful functionalities have been demonstrated from metamaterial-based devices, including negative refractive index components^4^, perfect absorbers^5^,^6^,^7^,^8^, modulators^9^,^10^,^11^, and filters^12^,^13^,^14^,^15^. Metamaterial concepts have also been widely applied in developing active terahertz filters by means of optical, electronic, and thermal stimulation^15^,^16^,^17^,^18^,^19^,^20^,^21^,^22^,^23^. And optical tuning of metamaterials is very attractive due to its fast speed^16^,^24^,^25^,^26^.

Tunable filters are pretty interested due to its flexibility in controlling THz wave, being crucial in THz imaging, sensing, and communications^27^,^28^,^29^. In the present work, tunable reflecting THz bandstop filter based on chirped metamaterial structure will be demonstrated by numerical simulation. Bandstop filter is commonly used to selectively eliminate undesired information and suppress interfering signals. So far, many tunable bandstop filters based on metamaterials^17^,^20^,^21^,^22^,^23^, such as mechanically tunable bandstop filters^17^,^20^,^21^,^22^, dynamic modulator based on microfluidic metasurface^23^, and active plasmonic bandstop filters resulting from varying of the chemical potential of graphene^15^,^30^, have been proposed.

Our designed tunable chirped metamaterial structure combines the chirped structure and photoconductive material. Broadband absorption can be constituted by including multiple metallic bars with varying length in a single unit cell^5^,^31^. The working frequency and transmission of metamaterial resonator can be tuned by photoexcited carrier injections when the photoconductive semiconductors are incorporated as elements of metamaterial structures^16^,^32^,^33^. In this letter, the metal bars are concatenated to silicon bars with different lengths. The metamaterial structure can be attributed to interference theory. In the interference model, our designed metamaterial filter could be decoupled into two interfaces, with the bars array and the ground plane located at the two sides of the dielectric spacer. And light illumination could to be introduced to change the conductivity of the silicon bars. When increasing the conductivity of the silicon bars, the silicon can realize a gradual transition from dielectric to metal, leading to different reflection and transmission coefficients of the bars array. Therefore, the reflectivity, central frequency and bandwidth of the metamaterial could be tuned.

## Results

### Metamaterial filter with chirped structure

**Figure 1:**
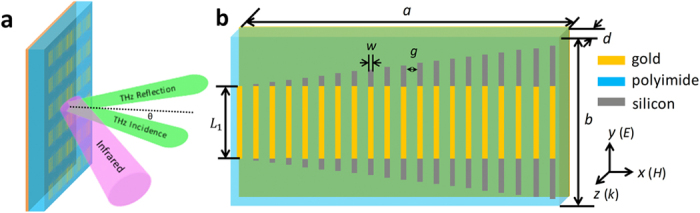
Schematic of optically controlled metamaterial terahertz bandstop filter. (**a**) A part of the metamaterial structure showing 6 × 3 unit cells. The metamaterial structure is devised by grouping thin hybrid bars together on a polyimide layer as a dielectric spacer, while the group plane is made of an opaque gold film. All the bars are centered in y-axis on the midline of each unit cell. (**b**) A unit cell of 20 bars with varying lengths (i = 1, 2……20) from 60 to 117 μm and equal spacing g = 10 μm. Each hybrid bar (except i = 1) in a single unit cell is composed of a metallic bar and two photoconductive silicon bars.

The tunable chirped metamaterial structure is inspired by two simple concepts that chirped structure and photoconductive material. In our design, the chirped structure refers to multiple metallic bars with varying length and silicon acts as the photoconductive material. By combing the benefits of the both concepts, we proposed a tunable metamaterial terahertz bandstop filter as shown in Fig. 1. Figure 1a displays a 6 × 3 unit cells’ array. Figure 1b describes one cell of the filter. A unit cell comprises 20 bars with varying lengths (i = 1, 2……20) and equal spacing. Each hybrid bar (except i = 1) in a single unit cell is composed of a metallic bar and two photoconductive silicon bars. The central yellow bars indicate golden bars and silicon (grey bars) is embedded in the both ends of each metallic bar. The silicon bars are different in lengths, while the widths and thicknesses are equal to those of the metallic bars. The filter consists of three layers: gold film substrate, polyimide (PI) spacer layer and metallic bars array, respectively. The unit cell is a = 300 μm in length and b = 120 μm in width. The thickness of PI layer is set to be d = 22 μm. The width w and thickness t of all gold bars are 5 and 0.3 μm, respectively. Silicon (grey bars) is embedded in the both ends of each metallic bar. The length L of 20 hybrid bars of varies from 60 to 117 μm with a step of 3 μm.

### Tunable reflectivity

The proposed chirped metamaterial structure performs as a reflective array. The incident THz wave with a polarization parallel to the metallic bars was denoted as the TE polarization. The simulations resulted in complex S parameters, from which we obtained the frequency dependent reflectance *R* = |S_11_|^2^. In our design, the transmission *T* is zero due to the Au ground.

The reflectivity of the designed metamaterials has been investigated numerically by using a commercial tool – CST Microwave Studio. In simulation, gold was modeled as a lossy metal with the conductivity is σ_Au_ = 4.561 × 10^7^ S/m, the spacer layer was taken as a lossless polyimide dielectric with ε_PI_ = 3.1, and the silicon was treated as a lossless dielectric material with ε_si_  = 11.68, but the conductivity σ_si_ is a pending value which could be controlled by light illumination according to different pump light powers. The dependence of the silicon conductivity on the pump power has been investigated in ref. 34. It is found that the conductivity is proportional to the pump power, but the conductivity should not be proportional to pump power at higher power, since carrier-carrier scattering resulting from a large carrier density saturates the conductivity and the probability of a multiphoton process is enhanced with the increase of pump power. Ref. 16 has indicated that a silicon conductivity value of 5 × 10^4^ S/m could be induced by near-infrared laser pulses with a center wavelength of 800 nm with average power of 500 mW (energy flux~500 μJ/cm^2^). Besides, an optical pump beam with an energy flux ~294.6 μJ/cm^2^ (@ 800 nm) was used to excite photoconductive silicon to obtain the conductivity of 1 × 10^5^ S/m^33^. Therefore, in our work, we hypothesis that the silicon conductivity could vary from 1 S/m to 1 × 10^5^ S/m.

A filter is generally characterized by parameters such as, modulation depth, center frequency, bandwidth and selectivity^35^. An important figure-of-merit is the modulation depth (MD), defined as MD = (*I*_*max*_ − *I*_*min*_)/*I*_*max*_, where *I*_*max*_ and *I*_*min*_ are the maximum and minimum reflectivity, respectively^36^. In practical filters, 3 dB bandwidth is defined as the difference in frequency between the half power points (*f*_*1*_ and *f*_*2*_) of the reflectance characteristic. The bandwidth of a filter gives information about its ability to separate components of similar amplitudes, and thus determines the resolution of the filter. The center frequency *f*_*0*_ is the arithmetic mean of *f*_*1*_ and *f*_*2*_. With respect to the filter, it should be mentioned that bandwidth is not the only factor determining the resolution capability. Selectivity is a descriptor which also indicates the ability of a filter to separate components of widely different levels. The basic parameter for selectivity is the shape factor, indicating the steepness of the filter characteristic outside the stopband, could be defined as the ratio of the filter bandwidth at an attenuation of 8 dB, to its 3 dB bandwidth^37^.

**Figure 2:**
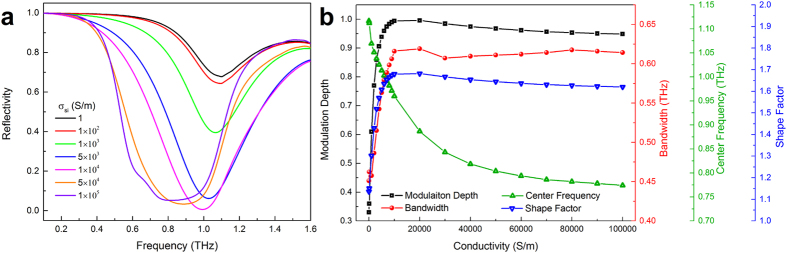
Tunable reflectivity. (**a**) Simulated reflection spectra of the bandstop filter for silicon with different conductivity. The curves are the reflectivity |S_11_|^2^ for the metamaterial structure shown in Fig. 1a. (**b**) The modulation depth, bandwidth, center frequency and shape factor of reflection spectra in Fig. 2a.

A numerical simulation was performed to analyze the reflection properties of the filter when the silicon had different conductivities. Figure 2a shows the reflection spectra from 0.1 to 1.6 THz for the silicon with several typical conductivities. One can see that the stopband shifts to a shorter frequency and the reflectivity in stopband decreases with an increase in conductivity. The concrete analysis of reflection properties is presented in Fig. 2b. Our results show that when the conductivity of silicon increases from 1 S/m to 2 × 10^4^ S/m, the modulation depth, bandwidth and shape factor will be added from 0.33 to 1, 0.45 to 0.62 THz, and 1.13 to 1.68, respectively, while the center frequency shifts from 1.12 to 0.88 THz. On further increase in the conductivity (2 × 10^4^ S/m^−1^ × 10^5^ S/m), the first three parameters remain about the same, but the center frequency has a slight variation of 0.11 THz. In addition, the modulation rate is limited by the recombination time (about 10 μs in our case) of the photo-excited carriers^38^, which can arrive 10^5^ Hz in principle. Based on the photoconductivity-induced mode-switching effect^32^,^34^, the device could work as a tunable broadband THz metamaterial bandstop filter.

### Angle-independent property

**Figure 3:**
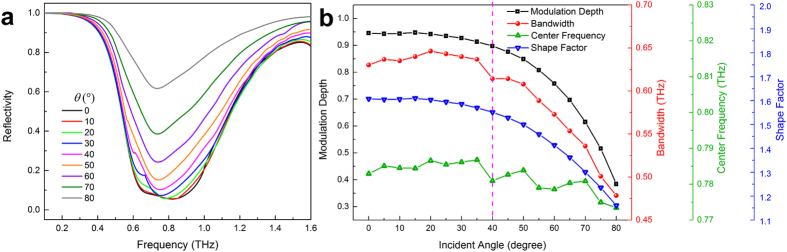
Angle-independent property. (**a**) The simulated reflection spectra for σ_si_ = 1 × 10^5^ S/m with incident angle *θ* scanning from 0° to 80°. (**b**) The modulation depth, bandwidth, center frequency and shape factor of reflection spectra in Fig. 3a.

Since the angle-dependent property is an important issue for practical applications, the simulated reflection spectra at different incident angles are investigated. The structure was simulated using frequency domain solver in CST Microwave Studio when σ_si_ = 1 × 10^5^ S/m. As shown in Fig. 3a, the reflectivity under oblique incidence angles begins to increase as the incident angle increases. Figure 3b presents the performance analysis of the tunable filter at different incident angle *θ*. We notice that the modulation depth, bandwidth, center frequency and shape factor of reflection curve remain almost constant for angles of incidence from 0° to 40°, which means that the reflection is insensitive to the incident angle. We also analyzed other conductivities, they all show angle-independent property in case of less than 40°. The loose angle tolerance allows the filter to be easily aligned.

### Polarization-dependent property

**Figure 4:**
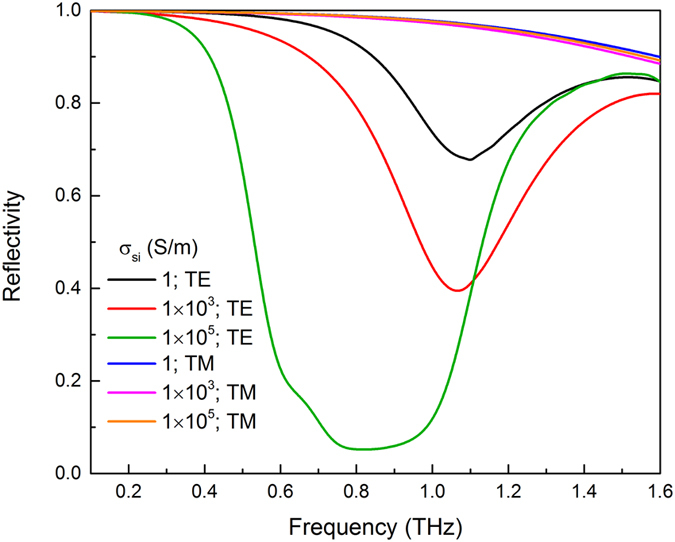
Polarization-dependent property. The simulated reflection spectra for both the TE and TM polarization incident waves when σ_si_ = 1 S/m, σ_si_ = 1 × 10^3^ S/m and σ_si_ = 1 × 10^5^ S/m.

The incident THz wave is TE polarization when the plane wave is polarized in the direction parallel to the bars, while the incident THz wave is TM polarization when the incident plane wave is perpendicular to the direction of the bars. The polarization used in this simulation is TE polarization. The reflection spectra have been achieved for the TE polarization, as shown in Fig. 2a. The stopband of the reflection spectrum shifts to a shorter frequency and the reflectivity in stopband decreases with an increase in conductivity. In Fig. 4, we also depicted three typical spectra for the TE polarization in order to compare with TM polarization when σ_si_ = 1 S/m, σ_si_ = 1 × 10^3^ S/m and σ_si_ = 1 × 10^5^ S/m. However, for the excitation with the TM polarization, the reflection spectra remain nearly constant. Apparently, the array is polarization-dependent, which can be utilized to modulate an incident TE polarization into a predefined reflective mode while maintaining normal reflection for the TM polarization.

## Discussions

We have proposed and numerically investigated a tunable THz broadband metamaterial reflecting bandstop filter based on metallic bars embedded with photoconductive silicon. Specifically, the modulation depth, bandwidth, center frequency and shape factor of the filter can be flexible by adjusting the conductivity of silicon. In general, when the conductivity of silicon varying from 2 × 10^4^ S/m to 1 × 10^5^ S/m, the four parameters of the filter can be tuned from 0.33 to 1, 0.45 to 0.62 THz, 1.12 to 0.88 THz, and 1.13 to 1.68, respectively. The mechanism to tune the stopband relies on manipulating bars array by changing the conductivity of photoconductive silicon. Furthermore, we could modulate the characteristic of the filter by changing the length and the number of bars in a single unit cell to tune bars array or varying the thickness of polyimide to tune the dielectric spacer. It could also be expected that if combined by structured light illumination, this chirped metamaterial structure could provide more freedom in controlling THz wave.

## Methods

### Sample design

**Figure 5:**
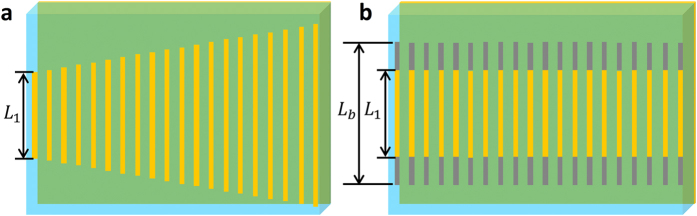
Two inspired models. (**a**) Model a: a unit cell of 20 metallic bars with different lengths varying from 60 to 117 μm. (**b**) Model b: a unit cell of 20 same hybrid bars (L_1_  = 60 μm, L_b_ is variable).

Model a shown in Fig. 5a and Model b shown in Fig. 5b show the examples of chirped structure and metallic bars structure embedded with photoconductive silicon, respectively. Figure 5a shows a unit cell of 20 metallic bars with varying lengths and equal spacing. Figure 5b displays a unit cell of 20 hybrid bars with same lengths and equal spacing, and each hybrid bar is composed of a metallic bar and two photoconductive silicon bars.

**Figure 6:**
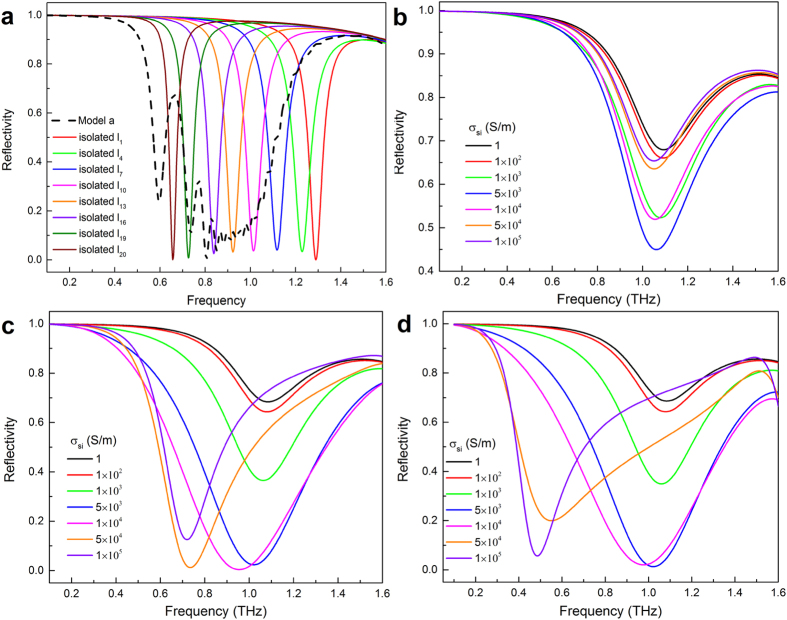
Reflection characteristics for Model a and Model b. (**a**) The curves are the reflectivity |S_11_|^2^ from the metamaterial structure of Model a and several isolated structures when i = 1, 4, 7, 10, 13, 16, 19 and 20. Simulated reflection spectra of the Model b for silicon with different conductivity when (**b**) L_b_ = 63 μm, (**c**) L_b_ = 90 μm and (**d**) L_b_ = 117 μm.

Model a is presented to interpret the physic mechanism of the broadband reflection in this work. Moreover, as shown in Fig. 6a, we provide the reflectance spectra in the situation of only having an isolated bar of Model a. The reflectance spectra are all narrowband and their peaks shift from 1.30 THz to 0.65 THz with length increasing from 60 to 117 μm, as shown in Fig. 6a. When the resonant frequencies are sufficiently close to each other, the broadband reflection (black dotted line in Fig. 6a) can be formed by including multiple metallic bars with varying length in the single unit cell as indicated in Model a. It’s worth mentioning that the narrowband peaks of isolated L_1_ and L_20_ shift slightly corresponding to the counterpart of Model a due to the unbalanced interactions among these bars. As a comparison, we also modeled an array with photoconductive silicon, as shown in Model b in Fig. 5b. Figure 6b,c and d indicate the reflectance spectra of Model b with different silicon conductivity when L_b_ = 63 μm, 90 μm, and 117 μm, respectively. The spectra feature will be modified with the silicon conductivity increasing from 1 S/m to 1 × 10^5^ S/m, but the trends of variation are inconsistent for different length of hybrid bars L_b_. The bars array of Model b is not only connected with silicon conductivity, but also related to the length of hybrid bars L_b_.

Furthermore, when the lengths of 20 bars in Model a are the same, the Model a is transitioned to Model b with silicon conductivity of 1 S/m (or without silicon). When the silicon conductivity reaches an order of 10^5^ S/m (gold conductivity is 4.561 × 10^7^ S/m), it can be regarded as metal. Therefore, when the conductivity of silicon increases from 1 S/m to 1 × 10^5^ S/m, the metamaterial filter with bars array could be recognized as making the transition from the Model b without silicon to Model a.

### Interference theory

**Figure 7:**
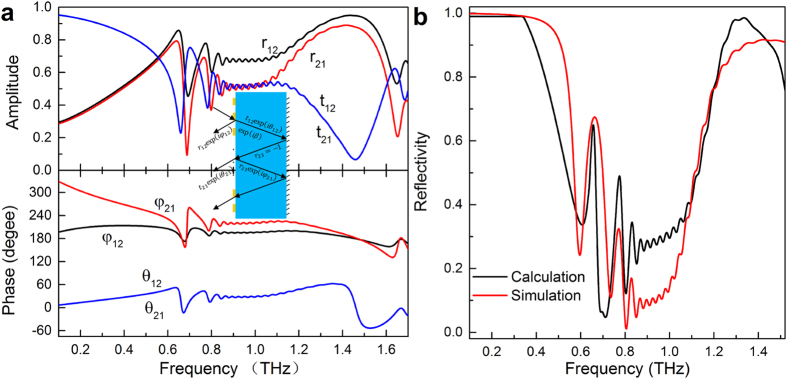
Interference theory explaining decoupled system of Model a. (**a**) Amplitude and phase of the reflection and transmission coefficients at the air-spacer interface with bars array. Inset: Multiple reflections and interference model. (**b**) The calculated reflectivity of the decoupled model a using interference model and the simulated reflectivity when treating the Model a as a coupled system.

Previous works have pointed out that the reflection peak mainly originates from the destructive interference of multi-reflection process between the metallic bars and the gold substrate^31^,^39^,^40^. The multilayered metamaterials can be divided into several individual layers without inter-layer resonance coupling and such individual layer properties are responsible for the overall properties^16^,^41^. Multiple reflections between the bars array and ground plane are shown in the Fig. 7a inset. At the air-spacer interface with bars array, the incident light is partially reflected back to air with a reflection coefficient 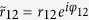 and partially transmitted into the dielectric spacer with a transmission coefficient 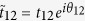. The latter continues to propagate until it reaches the ground plane, with a complex propagation phase 
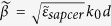
. After the reflection at the ground plane and addition of another propagation phase 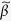, partial reflection and transmission occur again at the air-spacer interfere with coefficients 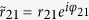 and 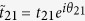. The overall reflection is the superposition of the multiple reflections:


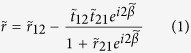


Taking Model a in Fig. 5a as an example, the reflection and transmission coefficients are shown in Fig. 7a, which can be obtained from simulating using Model a removed Au substrate. The reflectivity of Model a retrieved through 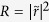 is described as the black line in Fig. 7b. The excellent agreement shown in Fig. 7b further validates the interference model.

## Additional Information

**How to cite this article**: Yang, J. *et al*. Tunable reflecting terahertz filter based on chirped metamaterial structure. *Sci. Rep.* **6**, 38732; doi: 10.1038/srep38732 (2016).

**Publisher's note:** Springer Nature remains neutral with regard to jurisdictional claims in published maps and institutional affiliations.
